# Anesthetic Selection for an Awake Craniotomy for a Glioma With Wernicke’s Aphasia: A Case Report

**DOI:** 10.7759/cureus.23181

**Published:** 2022-03-15

**Authors:** Heather Brosnan, Maranatha McLean, Apolonia E Abramowicz

**Affiliations:** 1 Anesthesiology, Westchester Medical Center/New York Medical College, Valhalla, USA; 2 Anesthesiology, University of Texas Southwestern Medical Center, Dallas, USA

**Keywords:** gleolan, 5-aminolevulinic acid fluorescence, neuromonitoring, bispectral index, awake craniotomy, neuroanesthesiology

## Abstract

Awake craniotomies for tumor resections allow for the preservation of eloquent cortex; however, they are high-risk surgeries that require careful patient selection and meticulous anesthetic management. Patients with significant preoperative language deficits may be unable to participate in intraoperative language mapping, increasing the risk of a failed surgery. Furthermore, anesthetic agents given for sedation and analgesia during the initial portion of the surgery may exacerbate existing language deficits. We present a case of an asleep-awake-asleep craniotomy for a left temporal lobe glioma using intraoperative neuronavigation, 5-aminolevulinic acid fluorescence, and awake speech mapping for a patient with a significant preoperative language deficit, for whom sedation had to be meticulously titrated to optimize intraoperative language testing. Anesthetic titration was aided by bispectral index monitoring, ultimately allowing successful awake speech mapping and tumor resection.

## Introduction

Preoperative patient selection is critical to a successful awake craniotomy because patients must be physically and psychologically capable of being safely kept awake for intraoperative cortical mapping and continuous language monitoring [1]. Patients with severe preoperative speech and language deficits are often excluded because they cannot meaningfully participate during intraoperative language mapping [2]. Patients with mild to moderate speech and language deficits may be considered for an awake craniotomy but are at higher risk for a failed surgery [2]. Intraoperative anesthetic management of these patients is critical to avoid exacerbating their baseline deficit with sedatives or analgesics.

We present a case of an asleep-awake-asleep (SAS) craniotomy for a high grade left temporal lobe glioma using intraoperative neuronavigation, 5-aminolevulinic acid (5-ALA) fluorescence, and awake speech mapping for a patient with a profound preoperative language deficit, for whom sedation had to be meticulously titrated to optimize intraoperative language monitoring. Written authorization was obtained from the patient to publish this manuscript.

## Case presentation

**Figure 1:**
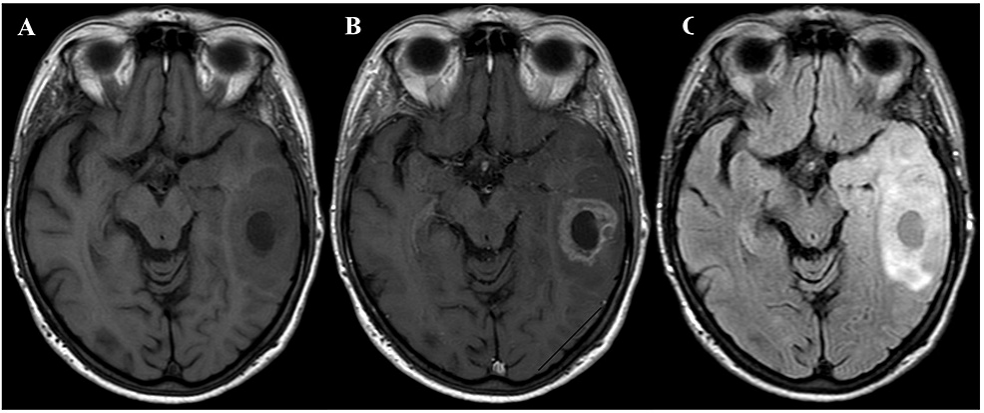
Preoperative MRI of the brain. Axial images of the brain showing a left temporal lobe glioma in close proximity to Wernicke’s area and the arcuate fasciculus, with significant vasogenic edema, and a 3-4 mm left to right midline shift. (A) T1 without contrast, (B) T1 with contrast, and (C) fluid-attenuated inversion recovery (FLAIR).

A 73-year-old, 50 kg woman with a history of diabetes, hypertension, anxiety, Hashimoto’s thyroiditis, osteoarthritis, and lumbar spinal stenosis presented with progressive short-term memory loss and word-finding difficulties. The patient’s neurological exam was notable for profound naming and word-finding difficulties, and altered sentence construction. An MRI demonstrated a 4 cm heterogeneously enhancing mass in the left temporal lobe, in close proximity to Wernicke’s area and the arcuate fasciculus, with significant vasogenic edema and a 3-4 mm midline shift (Figure 1). After initiation of dexamethasone, the patient underwent preoperative functional MRI, which demonstrated left language dominance. During this study, it was noted that the patient was significantly limited in verb generation, rendering portions of the exam non-diagnostic. Despite the existing language deficit, the neurosurgeon determined that an awake craniotomy with intraoperative language mapping, stereotactic navigation, and 5-ALA fluorescence would allow maximal tumor resection.

After a preoperative evaluation by the neuroanesthesiology team, a SAS approach with a supraglottic airway was determined to be the safest anesthetic option in the setting of both patient anxiety and intracranial mass effect. On the day of surgery, the patient received oral levetiracetam for seizure prophylaxis and intravenous dexamethasone for prevention of intraoperative nausea and vomiting. After an induction bolus of 60 mg of propofol, propofol and remifentanil infusions were started at 100 mcg/kg/min and 0.02 mcg/kg/min, respectively. A supraglottic airway was placed, and the patient was maintained on pressure support ventilation, maintaining end-tidal CO2 within normal limits. A scalp block was done by the anesthesia team using 26 mg of 0.5% bupivacaine to anesthetize the auricular temporal, greater and lesser occipital, supraorbital, supratrochlear, and zygomatic temporal nerves, bilaterally. The patient tolerated the block but was moving intermittently during this period. After receiving several additional propofol boluses, the propofol infusion was increased to 150 mcg/kg/min, and remifentanil was increased to 0.03 mcg/kg/min. Despite tolerating pinning without any hemodynamic changes, the patient continued to move intermittently. A bispectral index (BIS) sensor was secured to the forehead, with a reading in the high 60s to low 70s. A low dose dexmedetomidine infusion was started at 0.2 mcg/kg/hour due to concern that the patient may move after the Mayfield pin-holder was secured to the table, which decreased the BIS to the 50s.

With an adequate depth of anesthesia obtained, the neurosurgical team proceeded with stereotactic mapping and craniotomy. Meanwhile, over the course of approximately 30 minutes, the propofol and dexmedetomidine infusions were titrated down. Before the dura was opened, the sedation was turned off and the supraglottic airway was removed. Remifentanil was maintained at 0.02 mcg/kg/min for patient comfort. Continuous language testing was performed while the surgeon simultaneously mapped a grid around the tumor, stimulating at 9 mA and attempting to elicit speech arrest. The patient initially had difficulty with object naming tasks both with and without stimulation; however, as the patient awoke more fully from anesthesia, performance on these tasks improved. Furthermore, stimulation did not result in any positive findings, indicating that cortical areas superficial to the tumor did not contain any language function. As the neurosurgical team continued the resection, casual conversation with the patient was maintained, during which the patient endorsed hip and low back pain. Slight repositioning was attempted, and IV acetaminophen was given, but the patient ultimately required the addition of a low dose dexmedetomidine infusion at 0.2 mcg/kg/hour to tolerate the procedure. Approximately an hour into the awake portion of the surgery, as the surgeon resected the deeper borders of the tumor, the patient became fatigued. There were no specific deficits in language testing at this time, but the patient was intermittently falling asleep, preventing meaningful continued language monitoring. The patient had ingested 5-ALA preoperatively, which, in conjunction with stereotactic navigation, allowed the surgeon to continue additional tumor resection away from identified functional centers safely. The patient was then re-sedated with propofol and the supraglottic airway was replaced; the remainder of the surgery and emergence were uneventful. The surgeon estimated 95% tumor resection.

On immediate postoperative exam, repetition and object naming was intact, with only mild word-finding difficulties noted. The patient’s postoperative course was uneventful. Pathology was ultimately diagnostic for glioblastoma multiforme.

## Discussion

Awake craniotomies for tumor resections allow for the preservation of eloquent cortex and have been associated with improved overall and malignancy-free survival [1]. However, these are high-risk surgeries that require careful patient selection and meticulous intraoperative anesthetic management [1,3]. Patients who are unlikely to tolerate the awake portion of the procedure, such as those with high baseline anxiety, are often excluded [1]. Patients with respiratory disease, such as sleep apnea, may also be excluded due to concern for intraoperative respiratory failure with limited airway access [1]. From a neuropathological standpoint, patients with severe preoperative aphasias are excluded as they cannot participate in intraoperative language mapping [2]. Patients with mild to moderate aphasias may be considered but are at higher risk for a failed surgery [2].

An entirely awake craniotomy (AAA, awake-awake-awake) is an option for patients with preoperative speech and language deficits in whom sedatives or analgesics may worsen their baseline deficit [4]. Although local anesthetics are used to control pain caused by pinning, incision, and dural manipulation, patients may be unable to tolerate the discomfort of positioning and psychological distress of being awake for this portion of the surgery. Furthermore, severe hypertension can be catastrophic in patients with elevated intracranial pressure. While some evidence suggests that sedation for a SAS craniotomy may impact intraoperative language testing, this technique may be necessary for certain patients [5]. For example, in patients with mass effect or increased intracranial pressure, sedation and an invasive airway are often needed to hyperventilate the patient to decrease brain bulk, as was the case for our patient. Certainly, all patients receiving sedation for the initial portion of the surgery should remain either normocapnic or slightly hypocapnic because CO2 retention leads to increased brain bulk, resulting in brain herniation through the craniotomy once the dura has been opened. Given these risks, when a SAS approach is needed for a patient with preoperative aphasia, meticulous selection and titration of anesthetic agents become critical.

Dexmedetomidine, propofol, and remifentanil infusions are commonly used for sedation and analgesia during SAS craniotomies, and each drug has unique benefits and side effects. Dexmedetomidine is associated with better respiratory preservation, whereas propofol is associated with a decreased incidence of intraoperative seizures [6]. Remifentanil’s short duration of action makes it useful for intraoperative pain control and general comfort during both the asleep and awake portions; however, it increases the risk of nausea, vomiting, and respiratory depression [4]. Based on our literature review, no one has studied the individual effects of these agents on intraoperative speech and language mapping, so it is unknown whether there is a superior anesthetic for patients with aphasia undergoing SAS craniotomy [4,6]. Given that awake craniotomies are so often performed to preserve language centers, this remains a critical area of interest.

A handful of studies have assessed the utility of BIS monitoring during awake craniotomies. We used BIS because, despite a relatively high-dose propofol infusion, the patient continued to move, which can be particularly dangerous after the head has been secured in pins. The BIS aided in obtaining an adequate depth of hypnosis while using the minimum anesthetic dose possible, thus limiting the potential for prolonged sedation and exacerbation of the aphasia. While BIS value has been tracked during awake craniotomies, it is generally only employed as an adjunct monitor [7]. In a study by Mizota et al., pre-awake BIS values were not associated with somnolence (including the inability to assess speech function) during the awake phase; however, a secondary analysis of their data demonstrated that a BIS value ≤ 45 was associated with somnolence [8]. Maintaining a higher BIS value may improve patient participation in cortical mapping.

## Conclusions

Careful anesthetic selection and titration for patients with preoperative language deficits undergoing SAS craniotomies are essential for successful intraoperative language mapping; however, there are currently no studies comparing the effects of individual anesthetics on intraoperative language mapping in this patient population. Until more data are available to recommend a particular anesthetic agent, we recommend using the minimum dose necessary to achieve adequate sedation and analgesia. Furthermore, while BIS/processed EEG monitoring during the initial asleep portion of the surgery has not been definitely shown to improve intraoperative language testing, we recommend it as a tool to help guide anesthetic titration in patients who may be challenging to sedate and to help avoid excessively deep levels of sedation (BIS ≤ 45), which may improve participation in intraoperative language testing.
